# Sciuromorphy outside rodents reveals an ecomorphological convergence between squirrels and extinct South American ungulates

**DOI:** 10.1038/s42003-019-0423-5

**Published:** 2019-06-03

**Authors:** Marcos D. Ercoli, Alicia Álvarez, Adriana M. Candela

**Affiliations:** 1 Instituto de Ecorregiones Andinas (INECOA), Universidad Nacional de Jujuy, CONICET, IdGyM, Av. Bolivia 1661, 4600 San Salvador de Jujuy, Jujuy Argentina; 2 División de Paleontología de Vertebrados, Museo de La Plata, FCNyM, UNLP, CONICET, Paseo del Bosque s/n, 1900 La Plata, Buenos Aires Argentina

**Keywords:** Palaeontology, Palaeoecology

## Abstract

Notoungulates were a diverse group of South American ungulates that included the rodent-like typotherians. However, they are typically compared with other ungulates and interpreted as grazers. Here we present the first detailed reconstruction of the masticatory muscles of the pachyrukhine typotherians *Paedotherium* and *Tremacyllus*. An outstanding feature is the presence of a true sciuromorph condition, defined by an anterior portion of the deep masseter muscle originating from a wide zygomatic plate that reaches the rostrum, a trait traceable since the Oligocene pachyrukhines. Consequently, pachyrukhines are the first case of sciuromorph non-rodent mammals. This morphology would have allowed them to explore ecological niches unavailable for the exclusively hystricomorph coexisting rodents. This innovative acquisition seems to be synchronous in Pachyrukhinae and sciuromorph rodents and related to hard-food consumption. We postulate the expansion of nut and cone trees during the major environmental changes at Eocene−Oligocene transition as a potential trigger for this convergence.

## Abstract (web summary)

Marcos Ercoli et al. present a reconstruction of masticatory muscles in two species of Pachyrukhinae, showing evidence that these are the first known case of sciuromorph non-rodent mammals. They propose that this innovative acquisition occurred along with hard-food consumption.

## Introduction

The extraordinary ecological and evolutionary success of rodents is partially attributed to their particular, highly specialized masticatory apparatus with modified zygomasseteric structure and dentition adapted to gnawing and chewing^1–3^.

Sciurmorphy is defined as the extension of the deep masseter muscle onto the rostrum, which is attached to a widened and anteriorly tilted zygoma, i.e., zygomatic plate^3–5^. The sciuromorph condition is one of the three specialized forms (adding to histricomorphy and myomorphy) in which the rodents evolved to optimize the double function of gnawing with the incisors and grinding with the molars and premolars^3,6,7^. In the sciuromorph condition the lateral deep masseter muscle not only helps to advance the jaw for incisive occlusion, but also maximizes the power of the incisive bite^2,3,6–8^. Beyond the diverse dietary habits of sciuromorph rodents^2,5,9^, several contributions support that sciuromorphy arose as an optimization of the masticatory apparatus for hard object consumption^2,3,10^, and among living rodents, sciuromorph clades include some of the most specialized species for this feeding strategy^3,5,11^. Sciuromorphy occurred twice independently, in sciurids and castorimorphs (castorids and geomyoids^3,6,12^; and cites therein). The extinct species *Douglassciurus jeffersoni* (*Protosciurus jeffersoni*, late Eocene, 36 Ma^13^) is typically considered as the first sciurid, but contrary to later representatives of the family it has a protrogomorph zygomasseteric configuration, in which the deep masseter muscle originates from the anterior root of the zygomatic arch which is not expanded and does not reach the rostrum^4,5,13^. Sciuromorphy in sciurids arose during the *Grande Coupure*, in relation of the global cooling linked to the major climatic event of the Eocene−Oligocene transition^14,15^. The first record of a sciuromorph sciurid corresponds to *Protosciurus* (late Early Oligocene, 33–23 Ma^5,16,17^).

Among the faunistic elements that inhabited South America during the Cenozoic, there are the Typotheria, a diverse group of native ungulates. Within this clade, the Typotherioidea grouped Archaeohyracidae, Mesotheriidae, and Hegetotheriidae^18^. The first major diversification moment in which typotherioids became taxonomically and ecologically diverse was at the Eocene−Oligocene transition^18–20^. The families Mesotheriidae and Hegetotheriidae, recorded from the Early Oligocene, convergently acquired rodent- or rabbit-like morphologies^20–22^, presenting hypsodont dentition with chisel-like incisors, wide rostrum with diastemata, and several others cranial and postcranial features^18,19,22–29^. Among typotherioids, the group which more closely resemble rodents, by morphology and size, are the Pachyrukhinae, a Hegetotheriidae subfamily probably also recorded from the early Oligocene^30^ and whose biochron extends to Late Pliocene^26,31^.

A cranial feature that characterizes Typotherioidea^32^ is the presence of a zygomatic plate, which has been considered an unambiguous synapomorphy of this clade^18^. However, the functional and paleobiological significance of this structure and the overall morphology of the rostrum have been little studied. In this sense, there exist only some comments on the paleobiology of this extinct group and there are few ecomorphological studies regarding the cranial morphology of typotherioids^19,21,24–26,28,33–35^. Some few contributions that partially analyzed the masticatory muscle attachment areas, including the contributions of Cassini^34^ and Cassini and Vizcaíno^35^ for *Pachyrukhos moyani*, and Sosa and García López^36^ for *Paedotherium typicum*, stand out.

Beyond the fact that the zygomatic plate of typotherioids is at first sight similar to that of sciuromorph rodents, as was earlier stated by Patterson^25^ for some mesotheriids (see also refs. ^22,32^ versus ref. ^36^), the remaining contributions that described these notoungulates made reference to rodent-like morphologies in a broad sense, or compared them exclusively with caviomorph rodents, lagomorphs, hyracoids, and/or small ungulates. These studies lacked sciuromorph rodents as comparative models or even proposed alternative interpretations of the rostral anatomy^34^. As was stated by Gomes Rodrigues et al.^22^, there exists a necessity of more detailed studies of the morpho-functional feeding ecology of the masticatory apparatus to solve the relationship between the evolution of the rostral morphology of typotherioids and notoungulates and the potential drivers such as ecology and environmental conditions. According to these authors, this relationship cannot be understood from the data available so far.

In this contribution, we present, to our knowledge, the first, largely detailed 2D and 3D muscular reconstructions of Pachyrukhinae representatives in particular, and Typotherioidea in general. Following previous authors^2,3^, we hypothesize that sciuromorphy is optimized to gnaw hard objects. We propose that pachyrukhines would have reached a true sciuromorph zygomasseteric configuration, which would be interpreted in the same way. Additionally, we propose this morphology as a result of an ecological convergence with sciuromorph rodents linked to the global environmental changes at the Eocene−Oligocene transition, and discuss its evolutionary implications.

## Results

The reconstruction of the masticatory musculature in Pachyrukhinae revealed a distinctive morphology, with a remarkable large development and complex configuration of the masseter muscles. The reconstructed muscular mass of the mm. masseter (including zygomatico-mandibularis) represents 67.0% of the total masticatory muscles and 72.7% of the jaw-closing musculature (i.e., mm. masseter, temporalis, and pterygoideus) (Supplementary Table 1). These inferred values fit with typical proportions of rodents (masseter = 54–77% of jaw-closing muscles), and are specially similar to sciuromorph and hystricomorph masseteric models (59–76% for sciuromorphs, 57–82% for hystricomorphs^1,7,8,37,38^). Furthermore, they are larger than those of other rodents (58% for protrogomorphs, 54–64% for myomorphs^1–7^), lagomorphs (53–63%^1,39,40^), hyracoids (59%^41^), and any ungulate model (30–60% for ungulates; 40–58% for equids, 46–60% for bovids, 40–46% for cervids, 47% for giraffids, 30% for camelids, 42–50% for suids^1,40,42^). Although the musculature of fossil species can only be inferentially reconstructed, the abundant and well-preserved structures of the masticatory apparatus of fossil pachyrukhines, and the methods applied, allowed an acceptable inference (see Methods section).

### Zygomasseteric structure of Pachyrukhinae notoungulates

**Fig. 1:**
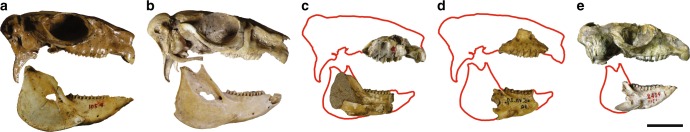
Best preserved cranial and mandibular remains of *Paedotherium* and *Tremacyllus* specimens studied. **a** *P. bonaerense* (cranium: MMP 1655-M, mandible: MACN Pv 10513–14), **b** *P. typicum* (cranium: MMP 1008-M, mandible: MLP 12-2703), **c** *P. minor* (cranium: MLP 29-IX-1-116, mandible: MLP 26-IV-10-37), **d** *P. borrelloi* (cranium: MLP 29-IX-1-116, mandible: MLP 26-IV-10-37), and **e** *T. impressus* (cranium and mandible of MACN Pv 2434). Scale bar: 20 mm

**Fig. 2:**
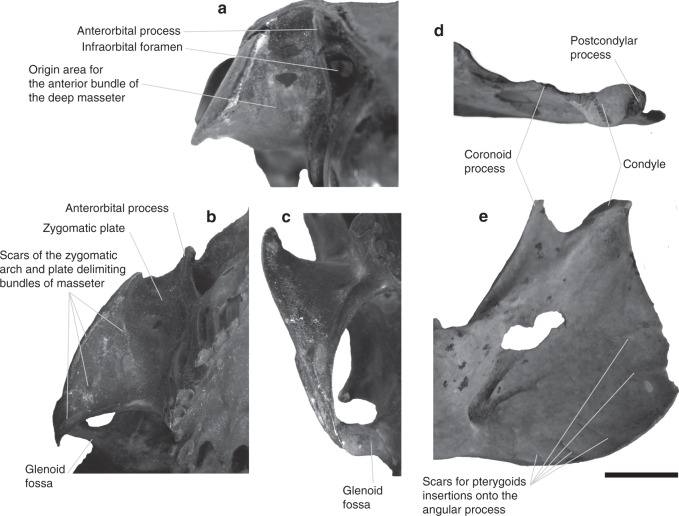
Anatomical details of skull anatomy of pachyrukhines. **a**−**c** Cranium of *Paedotherium bonaerense* MACN Pv 7253; **d**−**e** mandible of *P. typicum* MLP 12-2703. **a** Anterior view of zygomatic plate, **b** antero-ventral view of zygomatic plate and arch, **c** zygomatic arch and glenoid fossa, **d** dorsal view of condyle, and **e** medial view of ascending ramus. Scale bar: 10 mm

**Fig. 3:**
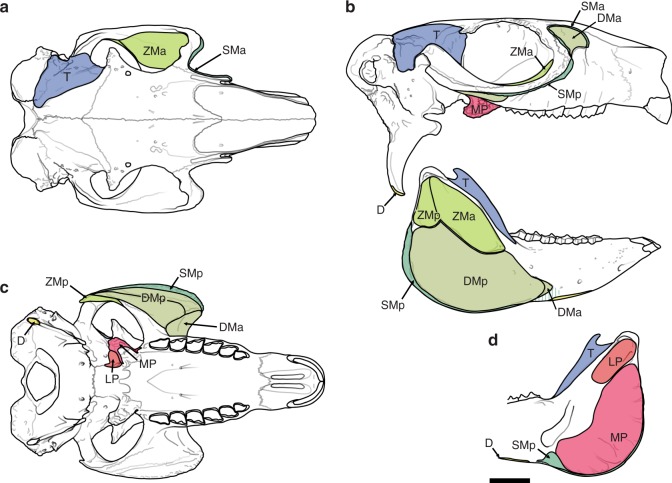
Muscular maps of *Paedotherium bonaerense*. **a** Dorsal view of cranium, **b** lateral view of cranium and mandible, **c** ventral view of cranium, and **d** medial view of mandible. D mm. digastricus, SMa and SMp anterior and posterior bellies of m. masseter superficialis (=superficial masseter), DMa and DMp anterior and posterior bellies of m. masseter profundus (=deep masseter), ZMa and ZMp anterior and posterior bellies of zygomatico-mandibularis, LP m. pterygoideus lateralis (=lateral pterygoideus), MP m. pterygoideus medialis (=medial pterygoideus), and T m. temporalis. Scale bar: 10 mm

**Fig. 4:**
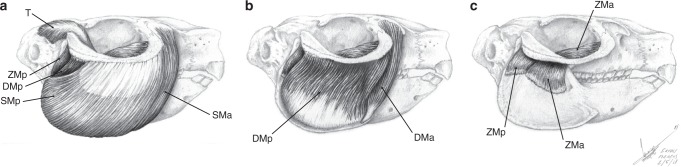
Muscular reconstruction of temporalis and masseter groups of *Paedotherium bonaerense*. **a** Superficial,** b** intermediate, and **c** deep layers. See legend of Fig. 3 for muscle abbreviations

In pachyrukhines (corroborated in *Paedotherium typicum*, *P. bonaerense* and *Tremacyllus*; Fig. 1a, b, e), the anterorbital process is delimited dorsally by a rough area, cranially ending as a spine, and caudally blending with the margin of the zygomatic arch, which is also rough (Fig. 2a−c). These marks are interpreted as the origin of the m. masseter superficialis (Figs. 3a−c, 4a; Supplementary Video 1) which would be wide and laminar in the extinct species. In basal forms, this spine is reduced or could be considered absent^18,26^ (see Supplementary Method), restricting the cranial extension of the muscle. The reconstruction of the m. masseter superficialis reaching the anterior spine of the anterorbital process agrees with the homology stated by Billet^20^ between the spine and the descending process of the zygomatic arch of other notoungulates (a structure also understood as the site of origin of the same muscle), and the origin reconstruction of that muscle for *P. typicum* of Sosa and García López^36^, although these authors split the spine origin from a secondary one restricted to the anterolateral margin of the arch. The laminar condition of the m. masseter superficialis is also present in *Heterohyrax*, as well as in several other mammals (e.g., perissodactyls, carnivorans); and it is considered as plesiomorphic for mammals^43^.

**Fig. 5:**
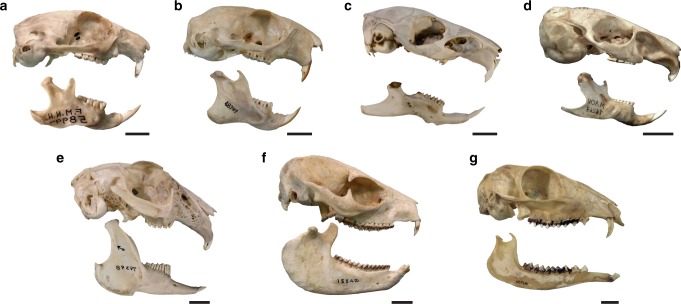
Representatives of the comparative sample of extant herbivore mammals. **a** *Cynomys ludovicianus* (FMNH 58999), **b** *Ratufa affinis* (FMNH 68747), **c** *Cavia aperea* (MMPMa ND 83), **d** *Chinchilla chinchilla* (MACN Ma 16267), **e** *Lepus capensis* (FMNH 79398), **f** *Heterohyrax brucei* (FMNH 18842), and **g** *Tragulus kanchil* (FMNH 68768). Scale bars: 10 mm

Another important evidence for the reconstruction of m. masseter superficialis as laminar in pachyrukhines is the absence of a circumscribed mark in the anterior root of the arch or in the latero-ventral surface of the rostrum (Fig. 1). In sciurids, as well as in some ungulates (e.g., *Tragulus*), the m. masseter superficialis originates from the latero-ventral surface of the rostrum, on a tuber (*Cynomys*), a rough ventrally positioned area (*Tragulus*), or an oblique ridge that anteriorly delimits the deep masseter muscle (*Ratufa*) (see refs. ^1^^,^^2^^,^^6^^,^^8^; personal data; Fig. 5a, b, g). This condition is a known convergence between both groups^43^. In *Lepus* and caviomorphs, the m. masseter superficialis also originates from a circumscribed mark, although located on the ventral aspect of the anterior root of the zygomatic arch, in a typically depressed area (see refs. ^44,45^; personal data). Neither of these two configurations corresponds to that present in Pachyrukhinae. Interestingly, some extant sciuromorph rodents possess both an extended laminar origin from the margin of the zygomatic arch and a restricted origin from the rostrum^8^; condition similar to the reconstruction proposed here (Fig. 4a).

The rough marks parallel to the margin of the angular process in the mandible correspond to the insertion site of a large part of this laminar muscle (Fig. 1a, b). They are also present in sciurids and *Lepus*, and secondarily in *Chinchilla* and *Tragulus* (Fig. 5a, b, d, e). They define the posterior limit between the insertion of the superficial and deep masseter muscles (Fig. 3b). *Heterohyrax*, as well as other hyracoids, display a distinctive morphology characterized by radial marks on the marginal sector of the lateral aspect of the angular process (Fig. 5f). They correspond to the insertion of a series of radial bundles, separated from each other by tendinous fascia, that belong to a well-developed m. masseter superficialis^41^. This does not occur either in other living species or in pachyrukhines, a reason for rejecting this arrangement of packages in fossils.

A bony structure of the mandible associated with the transit zone of m. masseter superficialis is a concave surface or groove located immediately anterior to the masseteric crest and its anterior process (when it is defined). This groove is only present in the rodent-like mammals analyzed, i.e., caviomorphs, *Lepus*, and sciurids (Fig. 5a−e); and the maximum extension is reached in *Cynomys* (Fig. 5a). When present, the groove contains and serves as a passage for a pars reflexa of the m. masseter superficialis^45^, which is also observed in all these rodent-like mammals (see refs. ^2,44,45^; personal data). On the other hand, the pars reflexa and its bony correlate are absent in cervids and hyracoids (see refs. ^1,41^; personal data). Cox and Baverstock^8^ described *Castor* as having no reflex portion, and the groove is accordingly absent in this taxon. In this context, its presence and development in pachyrukhines can serve as a reliable indicator of the presence of a pars reflexa of m. masseter superficialis (Figs. 3b, d, 4a; Supplementary Video 1), which would have a greater development in *Tremacyllus* than in *Paedotherium* species (Fig. 1).

The zygomatic plate, present in all typotherioids^32,46^, is particularly well developed among Pachyrukhinae (*Paedotherium typicum*, *P. bonaerense*, and *Tremacyllus*, and partially preserved in *P. borrelloi* and *P. minor*; see ref. ^26^; Figs. 1, 2), in which it extends along the rostrum. The degree to which this structure invades the rostrum is non-significantly related to variation in size (Supplementary Tables 2, 3). The zygomatic plate displays distinctive muscular marks delimiting more or less well-defined areas (Fig. 2a−c), which tend to coincide with fascias that delimit packages of the deep masseter muscle in living species (m. masseter lateralis pars anterior of Woods^45^) (see ref. ^1^; personal data). The largest and most anterior of these marks is rounded and extended along the surface of the zygomatic plate, reaching anteriorly the ventral aspect of the anterorbital process, a configuration that restricts the lateromedial expansion of the infraorbital foramen (Fig. 2a; Supplementary Video 1). A remarkably similar condition, with all these elements, is also present in many sciuromorph rodents (e.g., *Ratufa*; Fig. 5b). The degree of development of these structures in *Tremacyllus* is very similar to that of *Cynomys*, while in *Paedotherium* it is still more developed, especially in *P. bonaerense* (Figs. 1, 2a−c, 5a). In sciurids, this wide surface corresponds to the origin of an anterior division of the deep masseter muscle, a diagnostic configuration of sciuromorphy^2,4–6^; and the same is inferred for pachyrukhines (Figs. 3b, c, 4b, Supplementary Video 1).

A posterior division of the deep masseter muscle (m. masseter lateralis pars posterior) is inferred as originating from the remaining ventral surface of the zygomatic arch (Figs. 3c, 4b; Supplementary Video 1). This surface is characterized by longitudinally arranged marks (Fig. 2b, c), which could indicate the presence of aponeuroses separating muscle bundles.

With regard to the insertion of the deep masseter muscle, the mandibular masseteric crest (or ventral masseteric crest, see below) and the associated fossa just above it are well-defined in pachyrukhines (Fig. 3b), as in sciurids and *Lepus*^2,44^ (Fig. 5a, b, e). This crest ends anteriorly as a protruding process, the anterior tuber, corresponding to a strong attachment of the anterior fibers of the deep or superficial masseter muscles. The delineation of these structures is poorer or absent in *Heterohyrax*, *Tragulus* and the sampled caviomorphs (see refs. ^41,45,47^; personal data; Fig. 5c, d, f, g). Remarkably, the anterior tuber of the masseteric crest acquires an advanced position closer to the incisors than to the condyle in the pachyrukhines and especially in *P. bonaerense* (Figs. 1, 3b). This condition is known as a specialization of sciurids that maximizes the mechanical advantage of the deep masseter muscle for incisive bite, and is considered as part of the complex of features related to sciuromorphy according to Thorington and Darrow^2^.

The deepest masseteric layer in pachyrukhines would correspond, as typical in mammals in general, to the m. masseter zygomatico-mandibularis. Considering that it typically originates from a large part of the internal surface of the zygomatic arch in living taxa, it would have originated from the ventro-medial and medial surfaces of this structure (Figs. 3a−c, 4c). The insertion is on a well-defined dorsal fossa, present on the mandible of pachyrukhines (Figs. 1, 3b; Supplementary Video 1), as well as on that of sciurids and *Lepus* (Fig. 5a, b, e). In the specific case of caviomorphs, and in relation to their hystricomorph condition, there is an independent, large portion, denominated pars infraorbitalis that originates from the lateral wall of the rostrum and passes through the hypertrophied infraorbital foramen^43,45^ (Fig. 5c, d). A similar condition, although in reduced form, is also recorded in myomorphs^3^, but cannot be considered for pachyrukhines due to lack of the corresponding osteological features. It is worth mentioning that in caviid rodents (e.g., *Cavia*^45,48^), a dorsal masseteric ridge develops (Fig. 5c) in relation to the insertion of m. zygomatico-mandibularis bundles. This structure is not present in the other mammals analyzed here. The large extension of the ventral masseteric fossa in *P. bonaerense*, and secondarily, in *P. typicum*, would represent an increased area of insertion for the deep masseter muscle, in detriment of the insertion of the m. zygomatico-mandibularis. Conversely, the greater relative development of the dorsal fossa (although not surpassing the size of the ventral fossa) in *Tremacyllus* would indicate a greater relative importance of m. zygomatico-mandibularis (Figs. 1, 3b).

### Other masticatory muscles and temporomandibular joint

The skull of pachyrukhines, and especially in *Paedotherium bonaerense*, displays a very well-developed and rough pterygoid process and associated fossae, in a degree higher than the studied extant species. In the mandible, the shape of the angular process (the site of insertion of mm. masseter et pterygoideus) is very variable in mammals, mainly in relation to the configuration of pterygoideus (and in particular to m. pterygoideus medialis) than to the masseteric muscles. Among pachyrukhines, the contour of this process is wide and rounded, and it presents radial marks in its medial aspect (*P. typicum*, *Tremacyllus*, and especially marked in *P. bonaerense*; Figs. 1, 2e; Supplementary Video 1). These traits are also found in *Heterohyrax* and, secondarily, in *Tragulus* and *Lepus* (Fig. 5e−g), where the mm. pterygoidei are relatively enlarged^1,41,44^. In our comparative sample, *Heterohyrax* stands out for the greatest development of the angular process (Fig. 5f), while its shape and size are similar to that observed in pachyrukhines. In contrast, rodents possess a slender and caudally projecting angular process (Fig. 5a−d) with a medial pterygoideus shelf. The development of this shelf is lesser in caviomorphs than in sciurids, in which it is associated with radial marks in some representatives (e.g., *Cynomys*). This structure is moderately developed in *Lepus* (Fig. 5e). In pachyrukhines, the absence of a pterygoid shelf and the great development of the angular process with internal radial marks indicate a remarkable development of m. pterygoideus internus, with *P. bonaerense* showing the greatest development of this muscle (Figs. 1, 3d).

Furthermore, pachyrukhines possess a reduced postcondylar process associated with a small medial fossa (Figs. 1a, b, 2d, e, 3d). This condition is more likely linked to m. pterygoideus externus (Fig. 3d) than to a portion of the masseter muscle (e.g., posterior masseter of caviomorphs^49^), as is the case of *Lepus*.

In the cranium, the temporal fossae, site of origin of m. temporalis, are moderately extended (*Paedotherium typicum*, *P. bonaerense*, and *Tremacyllus*, Fig. 1a, b, e), being somewhat wider and reaching the middle line to form a sagittal crest in *Tremacyllus*^31^. In the mandible, the reduced coronoid process would be its exclusive insertion site (Figs. 1b, 3b, d). This morphology indicates a moderately to poorly developed m. temporalis, similar to that of *Cynomys*. Some divisions of this muscle are inferred through the presence of a marked ridge in the central area of the fossa (Figs. 1a, b, 3, 4a).

The glenoid fossa of pachyrukhines is an ample, concave and smooth surface, without pronounced lateromedial or anteroposterior limits (Fig. 2b, c). The condyle is slightly and uniformly convex and antero-posteriorly expanded, with its main axis tilting antero-laterally (Fig. 2d, e). This articulation is very similar to that observed in *Lepus*, and secondarily to that of the studied sciurids. Conversely, the condyle is very elongated, antero-posteriorly oriented, and restricted by a deep and narrow glenoid fossa especially in caviomorph rodents. Finally, *Heterohyrax* and *Tragulus* possess a transversely elongated condyle and glenoid fossa.

The morphology of the paraoccipital process of the pachyrukhines is different from all extant species, being markedly developed in the ventral direction, having a laminar appearance, and an inverted L shape (Fig. 1a, b). This morphology is probably associated with the hyoid apparatus. Although without evident marks, the mm. digastricus would originate, from the distal end of the paraoccipital process, as is the case for extant mammals (Fig. 3b), although with a relatively more ventral position than in our studied model specimens (Fig. 5). The anterior ventral surface of the mandible is smooth, as in *Heterohyrax* and *Tragulus* (see refs. ^1,41^; personal data), and no insertion marks or tubers are observed (Fig. 5f, g; Supplementary Video 1). Such marks occur in rodents and lagomorphs, usually represented by a tuber near the mandibular symphysis (see refs. ^1,2,44,45^; personal data; e.g., Fig. 5c).

## Discussion

The anatomical study of pachyrukhines reveals that they display a mosaic of traits, combining plesiomorphic characteristics, present in ungulates, with others, more derived and convergent with rodents. Interestingly, the informative osteological features analyzed here were well known since the late nineteenth century^23^, and have been used in recent systematic and evolutionary studies of notoungulates^20,22,26,31^. However, they had never been analyzed in a detailed morpho-functional way, like the one proposed here.

The presence of voluminous pterygoid muscles is linked to wide transverse movements for food grinding in ungulates. On the other hand, a posteriorly located insertion of mm. digastricus and a partly laminar m. masseter superficialis with a broad origin are plesiomorphic eutherian features, also present in many ungulates^1,43,50^. In addition, several derived traits are present in pachyrukhines, such as the great development and complexity of the masseteric musculature (dominating over other masticatory muscles, Supplementary Table 1), with an anterior portion located forward onto the rostrum, the presence of a pars reflexa of m. masseter superficialis, and a mainly antero-posteriorly extended temporomandibular articulation (Figs. 2d, e, 3; Supplementary Video 1). The masseteric muscular mass of some ungulates is relatively large (e.g., equids and bovids, and especially in grazer taxa^1,42^). Although proportions reconstructed should be taken with cautious (see Methods section), the proportion inferred for the mm. masseter in pachyrukhines is remarkably higher than any ungulate model and fit with sciuromorph and hystricomorph rodents. All these characteristics are remarkable convergences with rodents and lagomorphs, and can be associated with increased forces during incisive occlusion and a larger component of anteroposterior movements, allowing the action of gnawing. These results also highlight the necessity of using rodent and rodent-like comparative models to understand the anatomy of pachyrukhines and typotherioids, as in this study.

We propose that the most astonishing of these modifications is the presence of a true sciuromorph condition, with a broad zygomatic plate that reaches the rostrum and gives rise to a rostral portion of the deep masseter muscle, in convergence with sciuromorph rodents. As in living sciuromorphs, the advancement of the origin of the deep masseter muscle of the pachyrukhines was accompanied by an advancement of the insertion on the mandible, represented by the anterior tuber of the masseteric crest, maximizing the force of incisive occlusion^2–9^. Furthermore, pachyrukhines have several snout modifications associated with sciuromorphy shared with many living sciuromorphs, such as a reduced and lateromedial compressed infraorbital foramen and an anterorbital process extended in the vertical plane, both features linked to a greater space for the rostral origin of the masseter^2,16,17^ (Figs. 1, 2a−c, Supplementary Video 1). This set of traits provides evidence for an amazing case of convergence towards a sciuromorph morphology and common masticatory functions between these two phylogenetically distant clades, whose starting point involved totally different ancestral morphologies.

It has been proposed that a markedly extended zygomatic plate on the ventral and anterior aspects of the anterior root of the zygomatic arch would have arisen at least twice independently in Typotherioidea, in the common ancestor of mesotheriine mesotheriids and hegetotheriids^18,20,22^. The morphological analysis of typotherioids and the available descriptions (Supplementary Method; see refs. ^18,20,25^) confirm that pachyrukhines would be the only lineage that reached a sciuromorph condition. The myological and functional implications of the extended zygomatic plate of typotherioids have been rarely interpreted and in diverse ways. Patterson^25^ interpreted this structure as the site of origin of the deep masseter muscle for some mesotheriids, and indicated that this reminded of sciuromorph rodents^25^. However, these similarities are superficial, given that the zygomatic plate would never have reached the rostrum in mesotheriids. This condition of mesotheriids and other typotherioid lineages could be considered as a proto-sciuromorph configuration, just as protrogomorphy is for rodents. Sosa and García López^36^ recently performed a reconstruction of the masseter origins of notoungulates (including *P. typicum*). For typotherioids, these authors assigned the arch portion that conforms the zygomatic plate as the origin of the deep masseter (coinciding with Patterson’s proposal), being this origin located posterior to the anterorbital process (when present) instead reaching and occupying its ventral aspect. In relation with this, Sosa and García López^36^ did not recognize a sciuromorph configuration in pachyrukhines; furthermore, they considered that similarities with rodents are somewhat superficial^36^. Reguero et al.^26^ interpreted the zygomatic plate as the site of origin of the deep and superficial masseter muscles, and they compared this morphology to that of grazing ungulates. Cassini^34^ and Cassini and colleagues^21,35,51^ interpreted the anterior sector of the zygomatic plate as the origin of m. masseter superficialis, opposing the sciuromorph model and the homologies proposed in this contribution. This muscle reconstruction does not pass the second step of our methodology (see Methods section), considering that, in pachyrukhines, the greatest extent of the origin marks (anterior sector of the zygomatic plate) would be found in those species with the smallest development of the transit or insertion marks (groove anterior to the tuber of the masseteric crest and margin of the angular process), and vice versa. This does not occur in the extant sample either. In the same way as Reguero et al.^26^, Cassini and colleagues^21,35,51^ compared typotherioids (including the pachyrukhine *Pachyrukhos*) to living ungulates, and concluded that the large development of the structures related to m. masseter (i.e., zygomatic arch and mandibular angle) in the fossil taxa would be directly related to a grazing specialization, although recognizing a nonperfect fit and limitations in their comparative sample^51^. Following these osteo-muscular analyses and the presence of hypsodont dentitions (traditionally linked to grazers^51,52^), the pachyrukhines and some other typotherids are usually interpreted as forms adapted to grazing across open environments^53^. However, the interpretation of all pachyrukhines as grazers does not explain their eventual presence in forested environments of humid and subtropical climate^54^, neither the extinction of the subfamily when grasslands expanded during Late Pliocene-early Pleistocene^53^. It is worth noting that high-crowned teeth is a plesiomorphic feature of pachyrukhines and probably of typotherians^18,46^, and there exist strong evidence that support the decoupling between the origin of hypsodonty and the consumption of grasses by native South American ungulates^52,55^. In contrast to some poor hypsodont or brachydont sciuromorph rodents, marked hypsodont dentition of some sciuromorph rodents allow them to exploit environments with the presence of grit, to include abrasive components in food items, or to enable tooth digging activities^56^. In fact, many fossorial mammals, and particularly rodent taxa, have simplified and hypsodont teeth^6,21,56^. Following this, the teeth morphology of pachyrukhines could be linked to the fossorial habits proposed for some of them (inferred from forelimb anatomy and paleocaves analyses^51,57,58^). Pachyrukhines present terraced cheek teeth with a simplified occlusal design^26,46,59^, and large marginal cusps in the labial and lingual surfaces of upper and lower teeth, respectively^26,31,60^. Although the pachyrukhine simplified occlusal design can be related to allometric tendencies^46^, the presence of large cusps does not fit with the typical morphologies observed in grazing mammalian taxa, which present occlusal surfaces that are flattened (e.g., equids, caviids) or almost flattened (e.g., most of artiodactyls, and secondarily lagomorphs)^50,61,62^. The pachyrukhine morphology would favor a component of mortar-and-pestle-like crushing, instead exclusively grinding action during chewing as expected for specialized grazers. Based on our new paleobiological interpretation, we suggest that the paleoecological-environmental factors that could have driven the evolution of typotherioids, and pachyrukhines in particular, should also be revised.

**Fig. 6:**
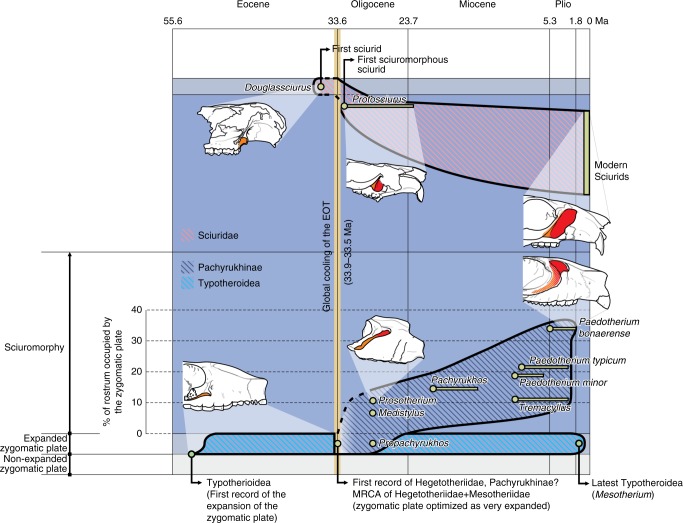
Summary outline about the morphological evolution of sciuromorphy in South American notoungulates (inferior region of the scheme) in comparison with that of sciurids (superior region of the scheme), indicating main related events on the time scale of the horizontal axis. Different background colors changing across the vertical axis indicates different morphological stages or conditions of the origin of the m. masseter lateralis (white, light blue and blue = non-expanded zygomatic plate, expanded but restricted to the zygomatic arch, and sciuromorphy = expanded and reaching the rostrum, respectively). Different conditions and convergence to sciuromorphy are illustrated by some pachyrukhine and sciurid representative. The degree of sciuromorphy was quantified (Supplementary Table 2) and plotted for pachyrukhines. Solid lines schematize the morphological diversity encompassed by each clade and known from fossil record, while dashed lines indicate main gaps in the fossil record. Biocrons of all pachyrukhines species with preserved rostrum, and of some representative fossil sciurids. Some of the information presented in this figure is taken from refs. ^17,18,20,26,30,60,78^

In their paleobiogeographic analysis, Seoane et al.^53^ proposed central Patagonia as the ancestral area of the Hegetotheriidae and Pachyrukhinae clades during the Deseadan age (late Oligocene, 29.4–24.2 Ma^63^), a moment dominated by warm climate. However, the earliest fossil record of these clades, and particularly of the latter, is incomplete. Hegetotheriids are well represented in the Deseadan age, with eight taxa that appear simultaneously and already morphologically diversified. Three are pachyrukhines, including *Medistylus* and *Prosotherium*^26,53,64^; which already display an established sciuromorph condition. Because of this, we think that the origin of pachyrukhines should be placed before these Deseadan fossils representatives. Furthermore, Reguero^30^ mentioned a fragmentary material of a hegetotheriid that would probably be a pachyrukhine from an Early Oligocene locality of Argentinian Patagonia (Cañadón Blanco, Chubut), supporting our statement. Although not preserved in the fossil record, the evolutionary leap, where the deep masseter muscle first reached the lateral aspect of the rostrum probably occurred at some time prior to Deseadan age. This condition was maintained or enhanced in all the later pachyrukhines, reaching the greatest development in the last representative of the group, *Paedotherium bonaerense* (Fig. 6; Supplementary Table 2).

Sciuromorphy and associated features have been deeply studied in living rodents, and it is known that this morphology optimizes the masticatory apparatus to bite and gnaw hard objects^2,3,6,7^. We believe that this dietary specialization, and not grazing, must have been the driver of the early evolution of the Pachyrukhinae. At that time, around the early Oligocene, the only rodents that inhabited South America were the caviomorphs which arrived to this continent in late Middle Eocene^65^ and reached southern latitudes in Early Oligocene^66^. The configuration of the hystricomorph model, a common feature of the representatives of this clade, is less optimal for gnawing hard objects than sciuromorphy, and more effective for grinding action^3^. Consequently, the acquisition of a sciuromorph condition in pachyrukhines would have allowed them to conquer ecological niches not attainable by caviomorphs.

While in South America the morphologies of the masticatory apparatus of the typotherians evolved from an ancestral ungulate morphotype and some approached (Fig. 6, light blue zone reached by Typotherioidea) or reached (Fig. 6, blue zone reached by Pachyrukhinae) a sciuromorph morphology, the acquisition of sciuromorphy occurred at the same time in rodents of Eurasia and North America, starting from a generalized protogromorph rodent morphotype (Fig. 6, blue zone). This evolutionary leap is well traced in the fossil record of sciurids^5,16,17^. The first sciuromorph sciurid appeared linked to the *Grande Coupure* event during the climatic deterioration of the Eocene−Oligocene transition, in which the paleobotanical community was altered, including the expansion of nut producers Fagales (Fagaceae, Betulaceae and Juglandaceae), and the diversification of nut morphology^14,15^. This event opened a new niche space and a mutualistic relationship between those rodents adapted to gnaw hard food and those nut trees^15,67^. Although less studied, the origin and first radiation of the other sciuromorph rodents, the Castorimorpha, was linked to the same climatic event^12,16,68,69^.

In South America, this global climatic event generated an analogous change in the vegetational community, in which the nut producers Fagales, Nothofagaceae (*Nothofagus*), expanded from Antarctica and invaded the southern South American forests, and the cone producers Podocarpaceae and Araucariaceae also expanded as the second floristic component of those forests (see ref. ^70^ and cites therein). The marked expansion of the zygomatic plate and the innovative acquisition of the sciuromorph condition seem to be a synchronous evolutionary process in both notoungulates and rodents, and occurred likely during the Eocene−Oligocene transition, a moment marked by major environmental, paleobotanical, and ecological changes. Previous morpho-functional analyses of sciuromorph rodents^2,3^ and our analysis of pachyrukhines allow us to postulate that these acquisitions are better understood as an adaptive convergence to hard food consumption. Interestingly, the available information of enamel microwear for some hegetotheriids, including *Pachyrukhos*^71^, indicates that some of them, and especially the pachyrukhine representatives, possess high numbers of pits and scratches. Although hard-item-based diet was not considered in this study, the microwear pattern is more similar to the pattern observed in rodents specialized in hard-item consumption, than those that feed primarily on grasses^72^. Although our ecomorphological analyses do not indicate the exact kind of hard items that could be consumed by an extinct mammal, the dominance of nut- and cone-producing trees renders them possible candidates that triggered this convergence. However, more evidence is needed to evaluate this and other potential ecological factors in future studies, considering that diverse selective pressures could explain similar morphologies^73^.

The detailed anatomical study of pachyrukhines allowed a better understanding of their paleobiology. This information, in conjunction with the evaluation of other evidence, allowed us to postulate new potential drivers for the evolution of South American native ungulates, and an amazing and exemplary case of adaptive convergence between two phylogenetically, morphologically and geographically distant mammalian lineages. Finally, the new interpretation about the biological role of these mammals further contributes to the understanding of how South American paleocommunities evolved and were restructured during the greatest climate change of the Cenozoic.

## Methods

### Sample and comparative descriptions

The detailed anatomical studies were carried out on fossil remains including 23 crania and 22 mandibles that belong to the Pachyrukhinae *Paedotherium typicum*, *P. bonaerense*, *P. minor*, *P. borrelloi*, and *Tremacyllus* spp. (Fig. 1, Supplementary Method). Materials and previous anatomical descriptions^18,24–26,31,33,35,74^ of these and other typotherians were also considered (the pachyrukhines *Pachyrukhos moyani*, *Medistylus dorsatus, Propachyrukhos ameghinorum,* *Prosotherium spp.*, the hegetotheriine *Hegetotherium mirabile*, the archaeohyracid *Archaeohyrax patagonicus*, the interatheriid *Interatherium robustum*, the mesotheriids *Trachytherus spegazzinianus*, *Typotheriopsis* spp., *Pseutotypotherium* spp., and *Mesotherium cristatum*; Supplementary Method). The anteroposterior length of the rostral portion of the zygomatic plate and the rostrum, and regressions between these variables, were quantified in order to assess the sciuromorphy degree (Supplementary Table 2) and allometric component (Supplementary Table 3). The supplementary information provides the data set generated and analyzed during the current study.

As comparative models of diverse modes of life or lineages of small-sized herbivorous mammals, several living eutherian mammals were considered, including caviomorphs (*Cavia aperea* and *Chinchilla chinchilla*), sciurids (*Cynomys ludovicianus* and *Ratufa affinis*), hyracoids (*Heterohyrax brucei*), lagomorphs (*Lepus capensis*), and small ungulates (*Tragulus kanchil*) (Fig. 5). Two or three specimens per species were studied (Supplementary Method). Information from own dissections and previous contributions on the osteology and myology of these and other mammals was also considered^1,2,41,44,45^.

### 2D and 3D muscular reconstructions

From this information, and the detailed and comparative assessment of the osteological traits linked to muscular attachments, a muscular reconstruction of the masticatory apparatus of *Paedotherium* and *Tremacyllus* was accomplished. Muscular maps on schemes of bones were made to illustrate origins and insertions (Fig. 3). In order to present clearly the muscular reconstruction and to evaluate its coherence considering the spatial distribution of the muscular masses and their inferred functions, we perform illustrations and 3D models of *Paedotherium bonaerense*, the pachyrukhine species considered as presenting the greater degree of sciuromorphy (Fig. 4, Supplementary Video 1). Starting from the muscular maps, each muscle of the masticatory musculature was reconstructed using plasticine in a very well-preserved skull (MACN Pv 7253), and weighed in a precision balance. A 3D model of the masseteric musculature was constructed through photographs of MACN Pv 7253 using the software ReCap (version 5.0.0.30) and ReCap Photo (version 19.0.0.38) of Autodesk.

### Comments about the reliability of muscular reconstructions

Beyond that a soft-tissue reconstruction is always an inferential issue, muscular scars are direct evidences of the presence of muscles, and, in some cases, the development of the latter can be estimated reliably from the former^75,76^. In this context, we want to highlight the confidence of our proposal. In order to evaluate alternative configurations, we followed the next three-step procedure. Firstly, a trait-to-trait qualitative analysis of studied specimen in a comparative framework of diverse living species allowed us establishing well-supported potential correspondences (homology hypotheses) between each muscle scars (i.e., direct evidence) and the most probable muscle attached to them (a first level of inference; see refs. ^75,76^). The second step was to check the correspondence or, at least, the absence of marked contradictions, in the development of the origin and insertion scars. Although it does not always occur, there exist usually a correlation between the development of muscles, and that of their origin and insertion scars^76^, and this can be in turn tested in extant models. If more than one group of homology hypotheses is being considered, the one that fits best in this sense should be chosen. The third step involves the 2D and 3D schemes and reconstructions that allowed us checking the topological and functional coherence of the whole set, re-analyzing the first and second steps when the reconstructed configuration differs from common features present in all living models (e.g., to reconstruct a muscle as deep, although this muscular layer is always superficial in living models), or when complex topological relationships without clear functional explanation arise (e.g., necessity of complex twisting of muscular mass to achieve a connection between the origins and insertions).

The assessment of the thickness of each muscular mass during the 3D reconstruction was made following the common pattern of the extant comparative sample (e.g., masseter mass reaches or barely laterally exceeds the lateral margin of the zygomatic arch), or that of the most similar extant models in the case of some specific shared features (e.g., proximal sector of the anterior portion of the deep masseter voluminous and encased between the anterorbital process and the zygomatic arch, and narrowing toward its insertion as in sciuromorph rodents, instead of flattened such in other taxa). As was stated by Blanco et al.^77^, the uncertainty of reconstructions increases from muscles with very bounded limits for their cross-section development (e.g., temporalis, anterior bundle of deep masseter) to poorly delimited or encased ones (posterior bundles of superficial and deep masseter). Considering that errors are multiplicative when volume proportions are estimated, the inferred proportions for poorly delimited bundles presented in the Supplementary Table 1 should be taken with caution. Given that the modeling was performed adding layer by layer of each subunit in the same model, the exaggeration in the volume of one masseter subunit, consequent of a poorly estimated bounded with another subunit, implied a compensating underestimation of the volume of the latter; so the establishment of this internal limit does not affect the proportion value of the total mm. masseter estimation (which in turn depends on the reliability of the establishment of the external limits of the total mm. masseter and the masticatory muscles as a whole). Due to the reconstruction of main masticatory muscles as a whole unit are more encased than many of their individual subunits, these calculations could be considered as a more suitable coarse-grain approach for general muscle proportions than the poorer delimited subunits.

### Reporting summary

Further information on research design is available in the Nature Research Reporting Summary linked to this article.

## Supplementary information

Supplementary Information

Description of Additional Supplementary Files

Reporting Summary

Supplementary Movie 1

## Data Availability

All materials studied are housed in mammalogical and paleontological collections. All of them, together with their collection label, are mentioned in the manuscript and Supplementary Information file. The datasets supporting the conclusions of this article are included in the Supplementary Information file.
