# Supplementary material for: Sciuromorphy outside rodents reveals an ecomorphological convergence between squirrels and extinct South American ungulates
Source: Commun Biol. 2019 Jun 3;2:202. doi: 10.1038/s42003-019-0423-5 (PMC6546766; doi:10.1038/s42003-019-0423-5)
Supplement: Supplementary file 1 — Supplementary Information [file 42003_2019_423_MOESM1_ESM.pdf]

Supplementary Method. Materials and bibliographic descriptions of pachyrukhines considered for muscular reconstructions, comparative sample, and institutional abbreviations.

Pachyrukhinae considered for muscular reconstructions

Paedotherium bonaerense: MACN A 1251-52, MACN A 7214, MACN Pv 18098-100; MLP 99-X-2-1; IDGYM s/n; MMP 158-S, 1655-M; refs. [1,2]
Paedotherium typicum: MACN Pv 5751, MACN Pv 6436, MACN Pv 10513; MLP 12-1782, MLP 52-IX-28-14; MMP 698-S, 1008-M: refs. [1,2]
Paedotherium borrelloi: MLP 57-X-10-21, MLP 57-X-10-62, MLP 57-X-10-88, MLP 57-X-10-142; ref. [2]
Paedotherium minor: MLP 26-IV-10-37, MLP 29-IX-1-116, MLP 29-IX-2-20, MLP 29-IX-2-102, MLP 29-IX-2-103, MLP 29-IX-2-157, MLP 55-IV-28-30, MLP 55-IV-28-82; MMP 464-M; ref. [2]
Tremacyllus spp.: FMNH P 14456, FMNH P 14465; MACN Pv 2434, MACN Pv 2913, MACN Pv 7207; ref. [2]

Comparative sample

Typotheria

Medistylus dorsatus: ref. [3]
Pachyrukhos moyanoi: FMNH P 13049, FMNH P 13053; refs. [4,5]
"Propachyrukhos ameghinorum": ref. [6]
Prosotherium spp.: FMNH P 13422; ref. [7]
Archaeohyrax patagonicus: ref. [8]
Hegetotherium mirabile: FMNH P 13194
Interatherium robustum: FMNH P 13224; ref. [9]
Mesotherium spp.: MMP 897-M, MLP 12-1776; ref. [10]
Pseudotypotherium spp.: MACN Pv 4430; MMP 323-M; ref. [10]
Trachytherus spegazzinianus: FMNH P 13281; ref. [10]
Typotheriopsis spp.: FMNH P 14452, FMNH P 14477; ref. [10]

Extant mammals

Cavia aperea: MACN Ma 27.7; MMP Ma ND 83
Chinchilla chinchilla: MACN Ma 45.11, MACN Ma 16267
Cynomys ludovicianus: FMNH 14964, FMNH 58999
Heterohyrax brucei: FMNH 18842, FMNH 104600
Lepus capensis: FMNH 42407; FMNH 79398; MACN Ma 26084
Ratufa affinis: FMNH 68746, FMNH 68747
Tragulus kanchil: FMNH 68768, FMNH 68778

Institutional abbreviations

FMNH P, FMNH – Palaeontological and mammalogical collections, Field Museum of Natural History, Chicago, Illinois, USA.
IDGYM – Palaeontological collection, Colección “Saturnino Iglesias” del Museo de Ciencias Naturales, Instituto de Geología y Minería, Jujuy, Argentina.
MACN A, MACN Ma, MACN Pv – “Ameghino”, Mammalogical and “Museum” collections, Museo Argentino de Ciencias Naturales, Buenos Aires, Argentina.
MLP – Palaeontological collection, Museo de La Plata, La Plata, Argentina.

MMP, MMP Ma – Palaeontological and mammalogical collections, Museo de Mar del Plata, Mar del Plata, Argentina.

Supplementary Table 1. Reconstructed muscular mass for masticatory muscles of *Paedotherium bonaerense*, computed from the plasticine model in MACN Pv 7253. % mass = percentage of mass in relation to all masticatory muscles. % mass without digastricus = percentage of mass in relation to jaw-clossing muscles, i.e., excluding mm. digastricus.

**Muscle**	**% mass**	**% mass without digastricus**
Digastricus	7.78%	-
Pterygoideus	20.03%	21.72%
Temporalis	5.16%	5.60%
Masseter	67.02%	72.68%
Masseter superficialis, principal	16.73%	18.15%
Masseter superficialis, reflexa	6.13%	6.64%
Masseter profundus, posterior	16.35%	17.73%
Masseter profundus, anterior	14.79%	16.04%
Zygomatico-mandibularis	13.02%	14.12%

Supplementary Table 2. Values of the ratio between the anteroposterior length of the rostral extension of the zygomatic plate and the anteroposterior length of the rostrum (both measures taken from the anterior margin of the zygomatic arch) for a sample of sciuromorph Pachyrukhinae (N = 25). Mean values for each taxa were plotted in figure 6. For *Prosotherium "triangulidens"* an approximate value (*) is given because of the rostrum is not fully preserved in the specimen.

**Specimen**	**Ratio**
Paedotherium bonaerense	
IDGYM sn	37.50%
MACN A 1251 a 52	40.10%
MACN Pv 7253	30.00%
MACN Pv 10513-14	35.10%
MACN Pv 18098-100	35.10%
MLP 32-IX-27-39	36.10%
MLP 51-VI-11-4	33.70%
PVL 2272	37.60%
PVL 503	28.60%
Medistylus dorsatus	
MPEF-PV 693	6.70%
Paedotherium minor	
MLP 55-IV-28-30	18.20%
Pachyrukhos moyanoi	
AMNH 15743	16.50%
AMNH 9283	9.80%
FMNH P 13053	15.10%
Prosotherium "triangulidens"	
MACN A 12467 (52-464)	10.90%*
***Tremacyllus* spp.**	
FMNH P 14456	10.00%
MACN Pv 2434	11.60%
MACN Pv 6250	11.70%
MACN Pv 17547	11.10%
Paedotherium typicum	
MACN Pv 16752	19.40%
MACN Pv 17333	21.00%
MLP 12-1782	27.50%
MLP 52-IX-28-14	23.90%
MLP 91-IV-5-66	21.60%
PVL 3386	15.40%

Supplementary Table 3. Mean values of the rostrum length and ratio between both lengths of the zygomatic plate and rostrum for the analyzed typotherians with available data (N = 15; see Supplementary Appendix 1). Allometric component was assessed through ordinary regressions between these variables for sciuromorph Pachyrukhinae (i.e., excluding the type specimen of *"Propachyrukhos ameghinorum"*, see plate 1 of ref. [6]) and whole Typotheria analyzed. PAST[11] software was used to carried out these analyses. Explained variance (R^2) and significance (p-values) of the linear regression analyses are given.

**Taxon**	**Clade**	**Rostrum length**	**Ratio**
Archaeohyrax patagonicus	"Archaeohyracidae"	41.10	0
Hegetotherium mirabile	Hegetotheriinae	26.16	0
Interatherium robustum	Interatheriidae	24.60	0
Medistylus dorsatus	Pachyrukhinae	30.21	0.067
Mesotherium spp.	Mesotheriidae	67.82	0
Pachyrukhos moyanoi	Pachyrukhinae	24.85	0.138
Paedotherium bonaerense	Pachyrukhinae	30.88	0.349
Paedotherium minor	Pachyrukhinae	25.59	0.182
Paedotherium typicum	Pachyrukhinae	27.54	0.214
"Propachyrukhos ameghinorum"	Pachyrukhinae	28.00	0
Prosotherium spp.	Pachyrukhinae	36.40	0.109
Pseudotypotherium spp.	Mesotheriidae	62.32	0
Trachytherus spegazzinianus	Mesotheriidae	98.30	0
Tremacyllus spp.	Pachyrukhinae	20.38	0.111
Typotheriopsis spp.	Mesotheriidae	69.41	0
**Linear regressions**		**R^2**	**p-value**
Pachyrukhinae		0.009	0.841
Typotheria		0.193	0.101

References

1. Kraglievich, L. Sobre el conducto humeral en las vizcachas y paquirucos chapadmalenses con descripción del *Paedotherium imperforatum*. *Anales Mus. Hist. Nat. Buenos Aires* **34,** 45–88 (1926).
2. Cerdeño, E. & Bond, M. Taxonomic revision and phylogeny of *Paedotherium* and *Tremacyllus* (Pachyrukhinae, Hegetotheriidae, Notoungulata) from the late Miocene to Pleistocene of Argentina. *J. Vertebr. Paleontol.* **18,** 799–811 (1998).
3. Reguero, M. A., Dozo, M. T. & Cerdeño, E. A poorly known rodentlike mammal (Pachyrukhinae, Hegetotheriidae, Notoungulata) from the Deseadan (late Oligocene) of Argentina. Paleoecology, biogeography, and radiation of the rodentlike ungulates in South America. *J. Paleontol.* **81,** 1301–1307 (2007).
4. Ameghino, F. *Obras Completas y Correspondencia Científica de Florentino Ameghino* (comp. Torcelli, A. J.) 24 Vols. (Taller de Impresiones Oficiales del Gobierno de la Provincia de Buenos Aires, La Plata, 1853-1911).
5. Cassini, G. H. & Vizcaíno, S. F. An approach to the biomechanics of the masticatory apparatus of early Miocene (Santacrucian age) South American ungulates (Astrapotheria, Litopterna, and Notoungulata): moment arm estimation based on 3d landmarks. *J. Mamm. Evol.* **19,** 9–25 (2012).
6. Simpson, G. G. A Deseadan hegetothere from Patagonia. *Am. J. Sci.* **243,** 550–564 (1945).
7. Billet, G. Phylogeny of the Notoungulata (Mammalia) based on cranial and dental characters. *J. Syst. Palaeontol.* **9,** 481–497 (2011).
8. Reguero, M. A. & Prevosti, F. J. in *The Paleontology of Gran Barranca: Evolution and Environmental Change through the Middle Cenozoic of Patagonia* (eds Madden, R. H., Carlini, A. A., Vucetich, M. G. & Kay, R. F.) 148–165 (Cambridge University Press, Cambridge, 2010).
9. Sinclair, W. J. in *Reports of the Princeton University Expeditions to Patagonia, 1896-1899* (ed Scott, W. B.) 1–279 (Princeton University Press, Princeton, 1909).
10. Patterson, B. *Trachytherus*, a typotherid from the Deseado beds of Patagonia. *Field Museum Nat. Hist., Geol. Series*, **6,** 119–139 (1934).
11. Hammer Ø., Harper D. A. T. & Ryan P. D. PAST: paleontological statistics software package for education and data analysis. *Palaeontol. Electron.* **4,** 1–9 (2001).
