# Supplementary material for: Sciuromorphy outside rodents reveals an ecomorphological convergence between squirrels and extinct South American ungulates
Source: Commun Biol. 2019 Jun 3;2:202. doi: 10.1038/s42003-019-0423-5 (PMC6546766; doi:10.1038/s42003-019-0423-5)
Supplement: Supplementary file 2 — Description of Additional Supplementary Files [file 42003_2019_423_MOESM2_ESM.docx]

**Description of Additional Supplementary Files**

**File Name**: Supplementary Movie 1

**Description**: 3D models of the masseteric muscles of Paedotherium bonaerense (MACN Pv 7253). Blue = m. masseter superficialis; orange = m. masseter profundus, anterior belly; green = m. masseter profundus, posterior belly; yellow = m. zygomatico-mandibularis. The first sequence shows the "superficial layer", i.e., the model with all masseteric muscles, and the second sequence shows the "deep layer", i.e., removing the m. masseter superficialis.
