# Supplementary material for: Sciuromorphy outside rodents reveals an ecomorphological convergence between squirrels and extinct South American ungulates
Source: Commun Biol. 2019 Jun 3;2:202. doi: 10.1038/s42003-019-0423-5 (PMC6546766; doi:10.1038/s42003-019-0423-5)
Supplement: Supplementary file 3 — Reporting Summary [file 42003_2019_423_MOESM3_ESM.pdf]

natureresearch

Corresponding author(s): Marcos D. Ercoli

Last updated by author(s): 2019/04/03

Reporting Summary

Nature Research wishes to improve the reproducibility of the work that we publish. This form provides structure for consistency and transparency in reporting. For further information on Nature Research policies, see Authors & Referees and the Editorial Policy Checklist.

Statistics

For all statistical analyses, confirm that the following items are present in the figure legend, table legend, main text, or Methods section.

n/a	Confirmed	
☐	☒	The exact sample size (*n*) for each experimental group/condition, given as a discrete number and unit of measurement
☒	☐	A statement on whether measurements were taken from distinct samples or whether the same sample was measured repeatedly
☒	☐	The statistical test(s) used AND whether they are one- or two-sided<br>*Only common tests should be described solely by name; describe more complex techniques in the Methods section.*
☒	☐	A description of all covariates tested
☒	☐	A description of any assumptions or corrections, such as tests of normality and adjustment for multiple comparisons
☒	☐	A full description of the statistical parameters including central tendency (e.g. means) or other basic estimates (e.g. regression coefficient) AND variation (e.g. standard deviation) or associated estimates of uncertainty (e.g. confidence intervals)
☒	☐	For null hypothesis testing, the test statistic (e.g. *F*, *t*, *r*) with confidence intervals, effect sizes, degrees of freedom and *P* value noted<br>*Give P values as exact values whenever suitable.*
☒	☐	For Bayesian analysis, information on the choice of priors and Markov chain Monte Carlo settings
☒	☐	For hierarchical and complex designs, identification of the appropriate level for tests and full reporting of outcomes
☐	☒	Estimates of effect sizes (e.g. Cohen's *d*, Pearson's *r*), indicating how they were calculated

Our web collection on statistics for biologists contains articles on many of the points above.

Software and code

Policy information about availability of computer code

Data collection	tpsUtil and tpsDig were used to measure linear variables for index calculation. Autodesk softwares were used to built up the 3D models and video files.
Data analysis	Index calculation was carried out using Windows softwares. Past3 was used to make regressions.

For manuscripts utilizing custom algorithms or software that are central to the research but not yet described in published literature, software must be made available to editors/reviewers. We strongly encourage code deposition in a community repository (e.g. GitHub). See the Nature Research guidelines for submitting code & software for further information.

Data

Policy information about availability of data

All manuscripts must include a data availability statement. This statement should provide the following information, where applicable:

- Accession codes, unique identifiers, or web links for publicly available datasets
- A list of figures that have associated raw data
- A description of any restrictions on data availability

The original 3D model files generated are available from the corresponding author upon request. The measures´ dataset is in the supplementary file.

Field-specific reporting

Please select the one below that is the best fit for your research. If you are not sure, read the appropriate sections before making your selection.

☐ Life sciences ☐ Behavioural & social sciences ☒ Ecological, evolutionary & environmental sciences

Ecological, evolutionary & environmental sciences study design

All studies must disclose on these points even when the disclosure is negative.

Study description	The study was based mainly on qualitative analyses involving anatomical descriptions of extinct and extant species of mammals. Linear measurements of some cranial dimensions were taken to estimate an index that was illustrated in Figure 5 and shown in Supplementary Table 2. Size effect on this index was estimated through a regression (data and results in Supplementary table 3). Muscular reconstructions of masticatory muscles were made and illustrated through hand-made schemes and 3D models uploaded as Supplementary video files.
Research sample	We studied osteological materials (cranium and mandible) of several extant mammal species (rodents, lagomorphs, ungulates, hyracoids) and extinct species of Paedotherium and Tremacyllus and other typotherids.
Sampling strategy	While it was possible, we chose the most complete cranio-mandibular remains of extinct species. For the seek of comparison, we used two to three specimens of each extant mammal species that represent diverse mammal clades and habits.
Data collection	Digital photographs of specimens were taken by MDE and AA; linear measurements were taken by MDE on those digital images using tps software. Some of the images were given by colleagues, who were duly thanked. Some photos or images of bibliography were also used.
Timing and spatial scale	The collection of images began in August 2015. The museological collections are visited without a defined temporal outline.
Data exclusions	n/a
Reproducibility	Measurements taken: 1) length of rostrum, measured from the anterior root of the zygomatich arch to the anterior tip of nasal and 2) lenght of the origin of the anterior deep masseter, measured from the anterior root of the zygomatich arch to the anterior tip of the anterorbital process.
Randomization	There was no allocation of specimens into groups.
Blinding	Blinding was not relevant because there was no experimental design involved in the analyses presented.

Did the study involve field work? ☐ Yes ☒ No

Reporting for specific materials, systems and methods

We require information from authors about some types of materials, experimental systems and methods used in many studies. Here, indicate whether each material, system or method listed is relevant to your study. If you are not sure if a list item applies to your research, read the appropriate section before selecting a response.

Materials & experimental systems

n/a	Involved in the study	
☒	☐	Antibodies
☒	☐	Eukaryotic cell lines
☐	☒	Palaeontology
☒	☐	Animals and other organisms
☒	☐	Human research participants
☒	☐	Clinical data

Methods

n/a	Involved in the study	
☒	☐	ChIP-seq
☒	☐	Flow cytometry
☒	☐	MRI-based neuroimaging

Palaeontology

Specimen provenance	Access to specimens housed in paleontological collections was granted by the respective curators (L. Chornogubsky, MACN; M. Reguero, MLP; W. Simpson, FMNH; and M. Taglioretti, MMP). All studied materials (included those of literature) were originally extracted from several fossiliferous localities from Argentina, with ages from Oligocene to Pliocene.
Specimen deposition	For this study, we did not collected materials in the field; only museological ones were used.
Dating methods	No new dates were provided in this study.

☐ Tick this box to confirm that the raw and calibrated dates are available in the paper or in Supplementary Information.
